# Cardiorespiratory Fitness Is Associated with Executive Control in Late-Middle-Aged Adults: An Event-Related (De) Synchronization (ERD/ERS) Study

**DOI:** 10.3389/fpsyg.2016.01135

**Published:** 2016-08-03

**Authors:** Chien-Heng Chu, Kao-Teng Yang, Tai-Fen Song, Jen-Hao Liu, Tsung-Min Hung, Yu-Kai Chang

**Affiliations:** ^1^Graduate Institute of Athletics and Coaching Science, National Taiwan Sport UniversityTaoyuan, Taiwan; ^2^Department of Physical Education, National Taiwan Normal UniversityTaipei, Taiwan

**Keywords:** executive control, ERD, ERS, fitness, Stroop Test

## Abstract

The present study sought to determine whether cardiorespiratory fitness is associated with cognitive function in late-middle-aged adults from event-related desynchronization (ERD) and event-related synchronization (ERS) perspectives. Late-middle-aged adults were categorized into either the high-fitness group or the low-fitness group based on their estimated cardiorespiratory fitness values. The participants completed the Stroop Test, which is comprised of incongruent and neutral conditions, while the brain activities were recoded. The alpha ERD and ERS values based on the equation proposed by Pfurtscheller (1977) were further calculated. The results revealed that the adults with higher levels of cardiorespiratory fitness demonstrated superior Stroop performance, regardless of Stroop congruency. While these high-fitness adults had less positive upper alpha ERD values in the later epoch window compared to their lower-fitness counterparts, they had greater lower alpha ERD values in the early epoch window. Additionally, in the late epoch window, the high-fitness adults showed less positive lower alpha ERD values on neutral, but not incongruent condition, relative to their low-fitness counterparts. These findings suggest that cardiorespiratory fitness of the late-middle-aged adults is positively associated with cognitive functioning, especially the cognitive processes related to the inhibition of task-irrelevant information and those processes required the devotion of greater amounts of attentional resources to a given task.

## Introduction

As life expectancies have continued to grow, the size of the aged population has surged dramatically over the last several decades. As of 2010, 8% of the world's entire population was over 60 years of age, and it is estimated that that number will rise to ~21% by 2050 (United Nations. Department of Economic Social Affairs: Population Division, 2013). This rapid projected rate of population aging will bring increased susceptibility to non-communicable diseases (U.S. Department of Health and Human Services, 2010). Age-related impairments have also contributed to a wide variety of cognitive deterioration, such as diminished processing speed, reduced working memory, and poorer long-term memory (Park and Reuter-Lorenz, 2009). These aging-related disturbances lead to increased healthcare resource utilization, and as a result, there is increasing interest in exploring cost-effective strategies to deal with the relative issues (Williams and Kemper, 2010).

Fortunately, age-related cognitive decline is not entirely unavoidable. Higher cardiorespiratory fitness has been found to protect against such functional impairments in older populations (Angevaren et al., 2008; Boucard et al., 2012). Specifically, older adults with higher cardiorespiratory fitness have demonstrated better behavioral performances in terms of multiple cognitive tasks than those with lower fitness (Kramer et al., 2005; Prakash et al., 2011). The linkage between cardiorespiratory fitness and cognitive performance may involve a causal association, with enhanced cardiorespiratory fitness following exercise interventions having been found to lead to a variety of physiological structural changes that are related to cognitive functioning in late-middle-aged adults, suggesting that cardiorespiratory fitness is associated with enhanced cerebrovascular reserves for cognitive functioning (Angevaren et al., 2008; Erickson et al., 2008; Voss et al., 2013).

Interestingly, cardiorespiratory fitness is associated multiple aspect of cognitive processes, particularly cognitive processes involved executive control (Colcombe and Kramer, 2003; Colcombe et al., 2004). Executive control can be conceptualized as higher-order cognitive processes composed of a subset of processes, such as inhibition, planning, and switching (Miyake et al., 2000; Jurado and Rosselli, 2007). After analyzing 18 controlled clinical studies, Colcombe and Kramer (2003) reported that fitness training leads to improvements in all aspects of cognitive functioning in older adults; however, they found that tasks requiring executive control were more substantially improved (*ES* = 0.68) than those involving speed, visuospatial-awareness, and controlled cognitive functions. Colcombe et al. (2004) further examined the association between cardiorespiratory fitness levels, executive control, and volumes of prefrontal and parietal cortices through magnetic resonance imaging (MRI) approach. They observed that individuals with higher levels of cardiorespiratory fitness exhibited superior behavioral performance, in addition to exhibiting higher density in the brain regions such as the prefrontal and the parietal cortices involved in executive control (Colcombe et al., 2004). Similarly, another study employing functional MRI found that increased cardiorespiratory fitness following a 12-month exercise intervention was associated with improved executive control and more efficient brain functioning in the prefrontal, parietal, and sensorimotor cortical areas (Voelcker-Rehage et al., 2011). These findings suggest, from behavioral and neuroimaging perspectives, that cardiorespiratory fitness serves a protective function with regard to multiple aspects of age-related cognitive decline, including executive control.

Electroencephalography (EEG) has also been employed to explore cognitive functioning because it is noninvasive and can help shed light on the potential neural mechanisms underlying the executive control processes. EEG is believed to reflect spontaneous electrical activity in the brain that consists of diversified dynamic waveforms (Tong and Thakor, 2009). Among the brain activities measured by EEG, much attention has been attracted by alpha frequency, an oscillation ranging between 8 and 14 Hz that are associated with sensorimotor, psycho-emotional, memory, and attentional control (Basar and Guntekin, 2012; Bazanova and Vernon, 2014). Such alpha frequency activity has been linked to the underlying neuronal activities, and it has been suggested that alpha frequency activity is inversely associated with the level of cortical activity (Bazanova and Vernon, 2014).

Nonetheless, EEG studies examining both cardiorespiratory fitness and cognitive functioning have only been limited and indirect. Gutmann et al. (2015) examined whether both acute and chronic exercise affect resting state alpha peak frequency (iAPF) in healthy young adults. Their findings indicated that iAPF values were increased after acute exercise but not after 4 weeks of cycling exercise training. Hogan et al. (2013) evaluated the cognitive performance of high-fit and low-fit adolescents after the cessation of acute exercise by utilizing the EEG coherence analysis. Their findings revealed that both the behavioral and EEG coherence assessed after the cessation of acute exercise were moderated by the participants' fitness levels. Specifically, while the adolescents with higher fitness levels demonstrated faster response time after the exercise than after the rest condition, the adolescents demonstrated lower levels of alpha coherence in the resting condition for tasks requiring executive control, suggesting increased efficacy of the attentional system after the acute exercise for those with higher cardiorespiratory fitness.

Event-related changes in alpha frequency activity (ER%) have also attracted interest, with well-known measures including both event-related desynchronization (ERD) and event-related synchronization (ERS), i.e., the suppression or expression activity in the alpha frequency triggered by stimuli or events, which reflect decreases or increases, respectively, in the harmonization of a given neuronal population (Pfurtscheller and Lopes Da Silva, 1999). ERD and ERS have also been suggested to reflect, respectively, the release from inhibition and inhibition (Bazanova and Vernon, 2014). To the best of our knowledge, only one study has utilized ER% to explore the association between exercise, fitness, and cognitive performance in older adults (Chang et al., 2015), finding that older adults with higher fitness levels benefited more from acute exercise. That is, those with higher fitness levels demonstrated significantly faster response speed after the cessation of acute exercise than their counterparts with lower fitness levels. Interestingly, a greater alpha ERD value after acute exercise compared to the control condition was also observed, suggesting that increases in neural resources for attentional investment and top-down processes were induced by acute exercise.

Accordingly, higher cardiorespiratory fitness has been linked to executive control in older adults, with substantial evidence from both behavioral and neuroimaging perspectives having been established. Despite the fact that event-related brain oscillations provide efficient and higher time resolution in terms of investigating cognitive processes, no research involving the use of ER% value has specifically explored how cardiorespiratory fitness affects the cognitive functions in a late-middle-aged population. As such, the purpose of the current study was designed to examine the association between cardiorespiratory fitness and cognitive functioning as indicated by the alpha ER%. Specifically, cognitive functioning, as assessed by the Stroop Test that has been considered as susceptive measure for cognitive aging (Lague-Beauvais et al., 2013; Gauthier et al., 2015), cardiorespiratory fitness (Prakash et al., 2011; Gauthier et al., 2015), as well as neurophysiological indexes such as alpha ERD and ERS, were utilized. With respect to task performance, it would be expected that cardiorespiratory fitness might moderate the Stoop Test performance, such that late-middle-aged adults with higher cardiorespiratory fitness levels would demonstrate shorter response time or higher accuracy of the Stroop Test. With respect to neurocognitive processes indexed by the alpha ER%, it was predicted that late-middle-aged adults with higher cardiorespiratory fitness levels would demonstrated an increased alpha ERD or decreased ERS value compared to those with lower cardiorespiratory fitness levels.

## Methods

### Participants

Healthy male adults with ages ranging from 50 to 65 years old were recruited through flyers posted in a community center and invited to a laboratory located at National Taiwan Sport University, Taoyuan County, Taiwan. Potential participants were included if they met the following criteria: (a) right-hand dominant; (b) free from neurological disease, cerebrovascular disease, or psychoactive medication use; (c) normal or corrected-to-normal vision based on the minimal 20/20 standard; (d) no color blindness; (e) free of recreational smoking habit; (f) intact cognitive functioning, as assessed by the Mini-Mental State Examination (MMSE, scores > 24); and (g) able to perform exercise of moderate intensity without any potential risk, as assessed by the Physical Activity Readiness Questionnaire (PAR-Q). Each participant's physical activity level and working memory functioning were measured by, respectively, the International Physical Activity Questionnaire (IPAQ; Bauman et al., 2009) and the Digit Span test of the Wechsler Adult Intelligence Scale-Third Edition (Wechsler, 1997).

Each participant was then instructed to complete the submaximal cardiorespiratory fitness assessment and was categorized into either a high-fitness or low-fitness group based on his/her estimated maximal oxygen consumption (VO_2max_) value (i.e., higher or lower than the 40th percentile of the normative value of VO_2max_ for his/her age group; American College of Sports Medicine, 2013). Forty eligible participants (*n* = 20 for each group) were instructed individually to come to the laboratory again within a week, at which time they completed the behavioral and neuroelectric measures. The participants were each financially reimbursed $30 for their participation. All the participants provided informed consent, with the study itself having been approved by the Institutional Review Board of the National Taiwan Sport University. The participants' demographic and cardiorespiratory fitness characteristics data are presented in Table 1.

**Table 1:** **Demographic and cardiorespiratory fitness characteristics of the high-fitness and low-fitness groups (means ± SD)**.

**Variables**	**High-fitness (*n* = 20)**	**Low-fitness (*n* = 20)**
Age (years)	60.20 ± 4.07	58.70 ± 3.53
Height (cm)	159.32 ± 0.12	161.12 ± 0.10
Weight	60.43 ± 5.58	65.33 ± 6.45
BMI	23.74 ± 1.65	25.24 ± 3.12
Digit Span test		
Forward	11.50 ± 1.79	10.60 ± 2.11
Backward	5.60 ± 1.60	5.50 ± 0.95
VO_2max_ (ml/kg/min)	49.50 ± 10.66^*^	27.36 ± 4.50
IPAQ (MET/week)	3640.00 ± 2021.59^*^	1638.00 ± 2504.32

BMI, body mass index; MMSE, Mini-Mental State Examination; VO_2max_, EstimatedVO_2max_; IPAQ, International Physical Activity Questionnaire (MET).

* significant differences between groups, p < 0.05.

### Cardiorespiratory fitness measure

Each participant's cardiorespiratory fitness level, as indicated by the VO_2max_ measured in milliliters per kilogram of body-mass per minute (ml/kg/min), was estimated using the YMCA cycling ergometer protocol. The YMCA cycling ergometer protocol is a widely used submaximal cardiorespiratory fitness assessment for adults with Class A risk stratification (American College of Sports Medicine, 2013). The fitness assessment was conducted during the first visit to the laboratory, with the participants having been asked not to perform any strenuous exercise 24 h prior to the assessment. The given participant's heart rate was measured by a Polar Heart rate (HR) monitor fitted prior to the fitness assessment and was recorded at every 3 min throughout the fitness assessment.

The YMCA cycling ergometer protocol involves three or more 3-min stages of continuous cycling and is designed to increase the HR of participants to between 110 bpm and an HR that corresponds to 85% of the age-predicted maximal heart rate (i.e., 220 minus age). Briefly, participants were instructed to exercise at a pedaling rate and workload of 50 rpm and 25 W, respectively, in the initial stage. The HR during the last minute of the initial stage determines the workloads of succeeding stages. For instance, if a participant's HR in the last minute of the initial stage is between 80 and 89 beats/min, the workloads for second and third stages will be 100 and 125 W, respectively. The HR for the last 15 s of min 2 and min 3 at each stage were recorded. If these two recorded HRs differed by more than 5 bpm, an extra minute would be added until the participant's HR stabilized. The process was terminated when a participant's HR reached the steady-state, which was defined as being within 10 beats of 85% of the HR_max_. Two HRs recorded from the second to last stage and from the last stage, along with the participant's body mass, age-predicted maximum heart rate, and workloads from the last two stages, were employed to calculate the estimated VO_2max_ (American College of Sports Medicine, 2013). Additionally, the Borg Rating of Perceived Exertion (RPE) on a scale of 6–20 (Borg, 1982) was recorded every 3 min.

### Stroop test

The item-by-item computerized Chinese version of the Stroop Test conducted in the current study was built and recorded using the Stim^2^ software of the NeuroScan system (Neurosoft Labs, Inc. Sterling VA, USA). The Stroop task using Chinese characters has consistently induced a robust Stroop effect and has been utilized across a wide range of age groups (Liu, 2007; Wang, 2011; Wang et al., 2014). Two types of Stroop conditions were involved: the neutral and incongruent conditions. In the neutral condition, the stimuli consisted of colored squares printed in blue, red, or green color. The stimuli in the incongruent condition, on the other hand, consisted of three Chinese color-words [i.e., 
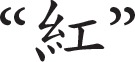
(red),
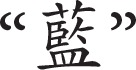
(blue), and 
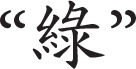
(green)] written in either of the two colors that did not match the semantic meaning of the given stimulus (e.g., the word “red” was printed in blue or green color). The stimuli proportions for each color of the colored square in the neutral condition were the same (e.g., 20 trials for each color of square), and so were the stimuli proportions for each possible color-word combination in the incongruent condition (e.g., 10 trials for each color-word combination). The stimuli in each block were presented in a randomized order.

All the stimuli were 2 cm in size and were presented in the center of white background displayed on a 15-inch computer screen with horizontal and vertical angles of 28.14° and 1.4°, respectively. Each stimulus was preceded by a fixation cross displayed in the center of the screen for 500 ms, after which the stimulus itself was shown for a maximum presentation duration of 500 ms.

Each participant practiced 12 trials prior to the formal experiment (i.e., 6 practice trials for the neutral condition and 6 practice trials for incongruent condition), having been instructed to respond, as quickly and accurately as possible, according to the chromatic nature of the stimuli by pressing, with the thumb of the left or right hand, one of three pre-specified buttons on the response box corresponding to each color. A response was accepted if it was made between 200 and 1000 ms after the onset of the stimulus. The response time (RT), defined as the time interval between the onset of a stimulus and a correct response made by the participant, and accuracy were subjected to further analysis.

### EEG recording and processing

Participants were instructed to be seated comfortably in a chair at a distance of ~65 cm from a 14″ computer screen in a sound and light-attenuated electrically shielded chamber. Throughout the recording of the EEG acquisition period, participants were instructed to keep their bodies as relaxed as possible. The EEG was recorded using the NeuroScan 4.5 system (NeuroScan Inc., El Paso, TX, USA). Prior to the administration of the Stroop Test, an electric cap (Neuroscan Quick-Cap, Neuroscan in. VA) with 32 Ag/AgCl electrodes arranged in the International 10–10 system (Chatrian, 1985) was mounted. Each electrode was referenced to the linked electrodes at the right and left mastoids with ground at FPz. The vertical and horizontal electro-oculographic (EOG) signals were simultaneously recorded from four electrodes placed below and above the left orbit and 1 cm lateral to the outer canthus of each eye, for the purpose of offline correction of ocular artifacts. For optimum signal transduction during the recording process, it is essential that a low resistance current path between the skull skin surface and a given electrode is provided. By filling the electrodes with the Electro-Gel^TM^ (Eelctro-Cap International, Inc., Eaton, USA) and constantly monitoring to ensure maintenance of the impedance value during the recording process, the impedance of all of the electrodes was maintained below 5 kΩ. The analog signal of the EEG was converted to a digital signal, amplified at a sampling rate of 1000 Hz (12 dB /octave), digitally low-pass filtered with 70 Hz and digitally high-pass filtered with 0.05 Hz (60-Hz note filter) by the NeuroScan SymAmp^2^ amplifier system (NeuroScan Inc., El Paso, TX, USA), and then stored to a hard disk on a continuous basis for off-line analysis.

The offline EEG data was further segmented into epochs ranging from −500 to 1000 ms relative to the onset of the stimulus using Editor 4.5 (NeuroScan Inc., El Paso, TX, USA) software. The trials with correct responses, but not those with incorrect responses or missing responses, were subjected to further analysis. After the data were recalculated based on the entire sweep serving as the baseline, as well as the EOG correction to remove the eye movement artifacts and eye blink activities, any trials for which exceeded ±100 μV were automatically rejected. In order to reduce leakage, a Hamming window of 10% length was also utilized. Following the procedure conducted by Cooper et al. (2013), two bands of event-related EEG activities, namely, the upper alpha (11–13 Hz) and lower alpha (8–10 Hz) bands, were computed using the Event-Related Bandpower transform in Neuroscan Edit 4.5. The Event-Related Bandpower transform permits intricate demodulation and simultaneous bandpass filtering (zero phase filter, 24 dB/Oct roll-off, envelope computed). Filter warmup artifacts at each end were trimmed by 300 ms from each end of an epoch.

The percent change in alpha frequency (ER%) was computed based on the equation proposed by Pfurtscheller (1977): ER% = (B–E)/B × 100, where the B denotes the mean alpha power (i.e., of upper alpha or lower alpha frequencies) during the reference interval (i.e., the time period 200 ms prior to the onset of a cue) and E denotes the mean alpha power during the period 0–700 ms after the onset of a cue. Positive and negative values for the ER% indicate, respectively, ERD and ERS alpha activities during the time epoch of interest in comparison with the reference period. The mean ERD/ERS values recorded for each of the three epoch windows (T1: 0–250 ms, T2: 251–500 ms, T3: 501–750 ms post-stimulus onset) were computed.

### Statistical analysis

The assumption of normality of data was assessed with the Kolmogorov–Smirnov test prior to the further statistical analysis. An independent *t*-test or chi-square test was conducted initially to analyze the means of the demographic and characteristic differences between the two groups in order to determine the appropriate assignment of the two groups.

The response times of correct responses as well as accuracy levels were separately computed by a 2 (fitness: high-fitness, low-fitness) × 2 (Stroop congruency: neutral, incongruent) mixed-model repeated ANOVA with Greenhouse-Geisser correction where appropriate.

The statistical analyses were confined to the Fz electrode location, with upper and lower alpha ER% values examined separately. Each of the three epoch windows (i.e., T1, T2, and T3) for the lower and upper alpha ERD measures were separately computed by a 2 (fitness) × 2 (Stroop congruency) mixed-model repeated ANOVA.

Findings of significant ANOVA effects were followed by multiple *t*-tests with Bonferroni adjustments. Greenhouse-Geisser corrections were utilized for the purpose of correcting for any violations of sphericity. Means and standard errors were presented. The family-wise alpha level was set at 0.05 for all statistical analyses.

## Results

### Participant characteristics

Descriptive data regarding the participants' demographic data are presented in Table 1.

No significant differences in age, height, weight, or BMI (*ts* > −1.2, *p* > 0.1) were observed between the two groups. Additionally, there were no significant differences between these two groups in terms of the raw scores of the Digit Span test and the MMSE (*ts* > −1.5, *p* > 0.1). With regard to cardiorespiratory fitness levels and physical activity levels, there were significant differences between the two groups for both VO_2max_ and IPAQ values (*t*s < −2, *p* < 0.01), suggesting that the participants were properly assigned into the two groups.

### Behavioral data

**Figure 1:**
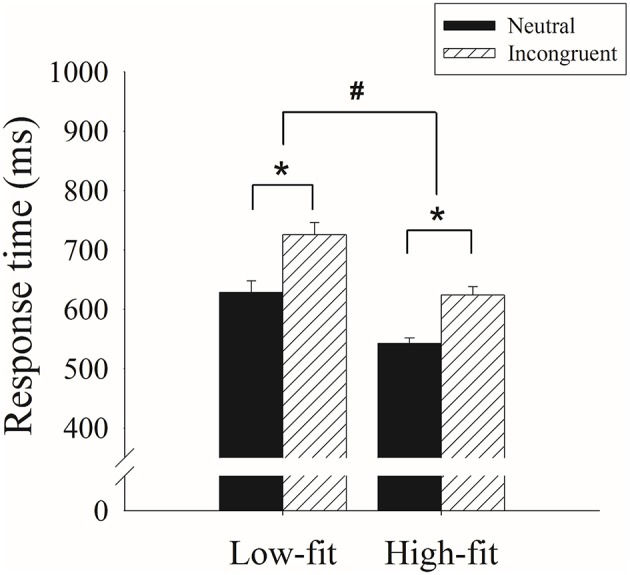
**Differences in the performances of the high-fitness (High-fit) and low-fitness (Low-fit) groups for the incongruent and neutral conditions of the Stroop Test**. ^*^represents the significant difference between Stroop congruency (*p* < 0.05). ^#^represents the significant difference between groups (*p* < 0.05).

The two-way ANOVA of response time revealed a main effect of group, *F*_(1, 38)_ = 17.42, *p* < 0.001, partial η^2^ = 0.31, with shorter response time on high fitness (583.55 ± 15.89 ms) compared to low fitness group (677.34 ± 15.89 ms). A main effect of Stroop conditions was also revealed, *F*_(1, 38)_ = 238.71, *p* < 0.001, partial η^2^ = 0.86, with longer response time on incongruent condition (675.16 ± 12.46 ms) compared to control condition (585.73 ± 10.68 ms; Figure 1).

The analysis of accuracy revealed only a main effect of Stroop congruency, *F*_(1, 38)_ = 28.58, *p* < 0.001, partial η^2^ = 0.43, with lower accuracy exhibited for the incongruent condition (0.90 ± 0.02) compared to the neutral condition (0.95 ± 0.01).

### ERD/ERS data

#### Upper alpha (11–13 Hz)

**Figure 2:**
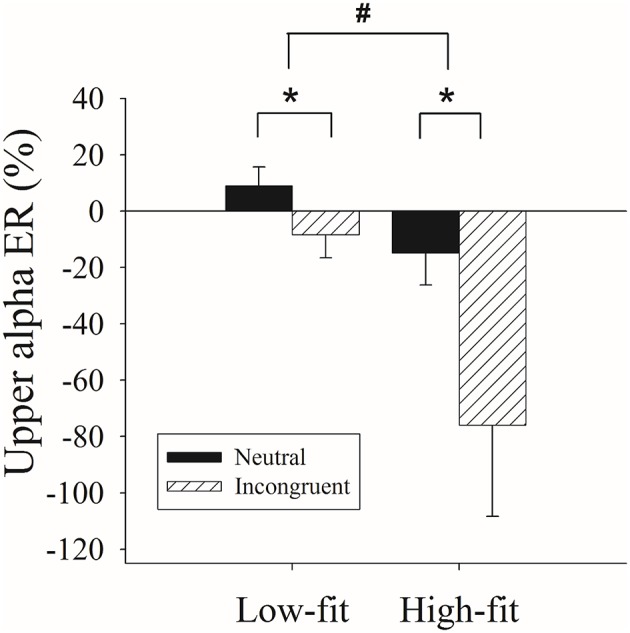
**Differences in the upper alpha frequency band (ER%) values for the T3 epoch window between the high-fitness (High-fit) and low-fitness (Low-fit) groups for the incongruent and neutral conditions of the Stroop Test**. ^*^represents the significant difference between Stroop congruency (*p* < 0.05). ^#^represents the significant difference between groups (*p* < 0.05).

The analysis of upper alpha ER% values for the T1 and T2 epoch windows revealed no main effects or interaction effects. The analysis of values for the T3 epoch window revealed a main effect of fitness, *F*_(1, 34)_ = 5.16, *p* < 0.03, partial η^2^ = 0.13, with less positive upper alpha ERD values for the high-fitness participants (−45.40 ± 15.00) than for the low-fitness participants (0.30 ± 13.42). A main effect of Stroop congruency was also revealed, *F*_(1, 34)_ = 11.63, *p* < 0.002, partial η^2^ = 0.26, with more upper alpha ERS values exhibited for the incongruent condition (−2.90 ± 6.30) compared to the neutral condition (−42.20 ± 15.14; Figure 2).

#### Lower alpha (8–10 Hz)

The two-way ANOVA of lower alpha ER% values for the T1 epoch window revealed a main effect of fitness, *F*_(1, 34)_ = 4.60, *p* < 0.04, partial η^2^ = 0.12, with greater positive lower alpha ERD values for high-fitness group (1.07 ± 3.38) compared to the low-fitness group (−8.65 ± 3.02). A main effect of Stroop congruency was also revealed, *F*_(1, 34)_ = 30.44, *p* < 0.001, partial η^2^ = 0.46, with less positive lower alpha ERD values exhibited for the incongruent condition (−13.06 ± 3.20) compared to the neutral condition (5.47 ± 2.38). However, the analysis of second time epoch revealed no any main and interaction effects.

**Figure 3:**
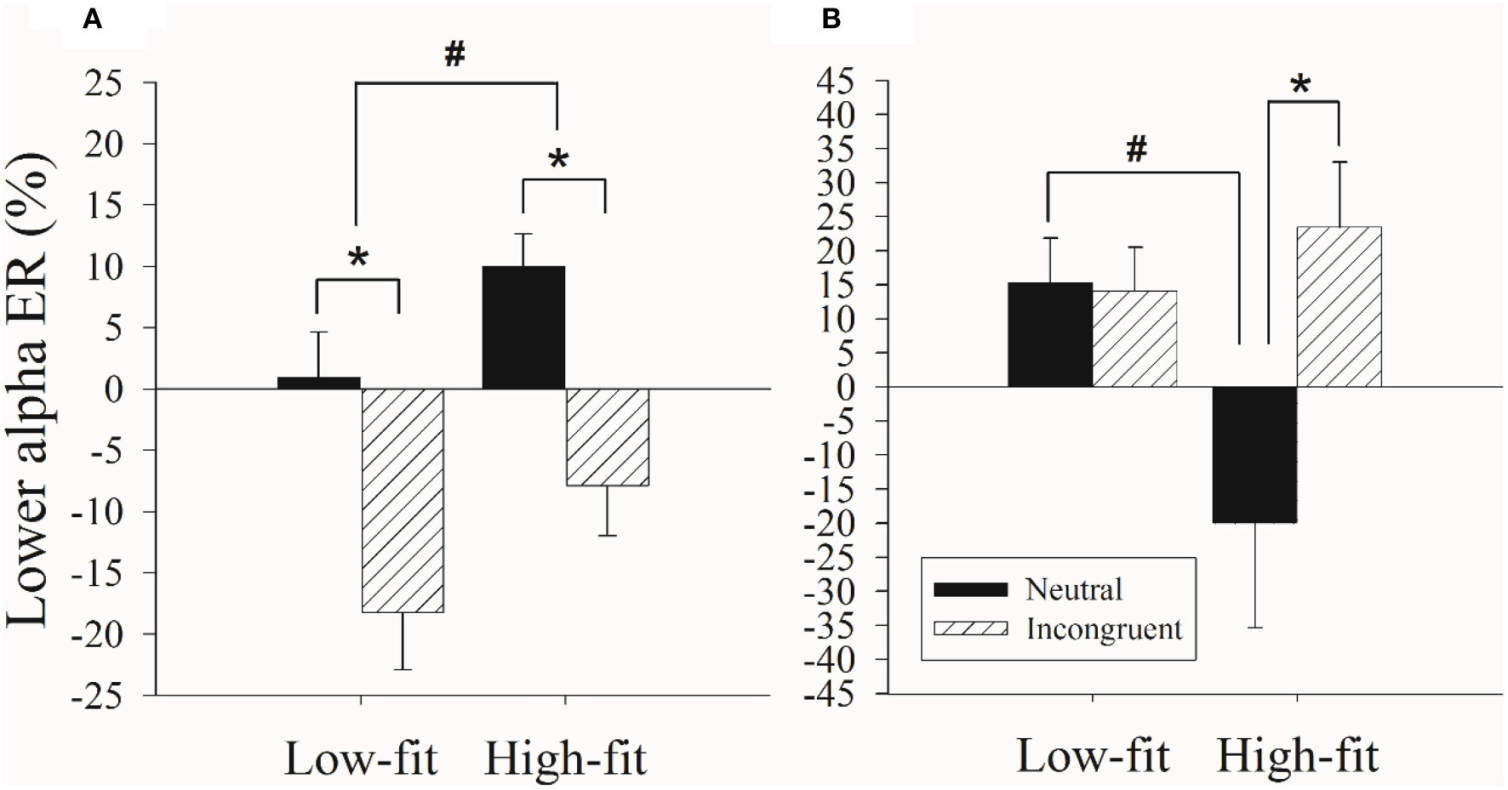
**Differences in the lower alpha frequency band (ER%) values for the (A) T1 epoch window and (B) T3 epoch window between the high-fitness (High-fit) and low-fitness (Low-fit) groups for the incongruent and neutral conditions of the Stroop Test**. ^*^represents the significant difference between Stroop congruency (*p* < 0.05). ^#^represents the significant difference between groups (*p* < 0.05).

Lower alpha ER% values for the T3 epoch window indicated significant effects of Stroop congruency [*F*_(1, 34)_ = 4.22, *p* < 0.05, partial η^2^ = 0.11], with the incongruent condition (18.73 ± 5.63) exhibiting greater positive lower alpha ERD values compared to the neutral condition (−2.39 ± 7.75). Additionally, a significant fitness × Stroop congruency interaction was also observed [*F*_(1, 34)_ = 4.74, *p* < 0.04, partial η^2^ = 0.12]. The follow-up analysis revealed that the high-fitness group exhibited less positive lower alpha ERD values (−20.08 ± 11.55) than the low-fitness group (15.30 ± 10.33) in the neutral condition (*p* < 0.03), but not in the incongruent condition (23.42 ± 8.39, 14.04 ± 7.50, *p* > 0.05). Additionally, the high-fitness group exhibited more positive lower alpha ERD values in the incongruent condition (23.42 ± 8.39) compared to the neutral condition (–20.08 ± 11.55; *p* < 0.01), while the low-fitness group exhibited no such difference (14.04 ± 7.50, 15.30 ± 10.33, *p* > 0.05; Figure 3).

## Discussion

Despite the fact that an increasing number of studies have examined the relationship between cardiorespiratory fitness and cognitive functioning, no research has directly investigated that relationship by utilizing ERD and ERS. Our primary behavioral findings revealed that late-middle-aged adults with higher levels of cardiorespiratory fitness demonstrated superior cognitive performance, as reflected by shorter reaction times in both congruency conditions of the Stroop Test, than those with lower cardiorespiratory fitness. Additionally, poor performances reflected by longer reaction time and less accuracy in incongruent condition were observed compared to those in neutral condition.

With regard to the upper alpha frequency, the high-fitness late-middle-aged adults exhibited less positive upper alpha ERD values in the later epoch window than their low-fitness counterparts. Additionally, the Stroop incongruent condition produced more upper alpha ERS values than the neutral condition. With regard to the lower alpha frequency, the high-fitness late-middle-aged adults exhibited greater lower alpha ERD values in the early epoch window. However, for the late epoch window, the high-fitness late-middle-aged adults showed less positive lower alpha ERD values in the neutral condition, but not the incongruent condition, relative to their low-fitness counterparts. Additionally, the high-fitness late-middle-aged adults showed more positive lower alpha ERD values in the incongruent condition compared to the neutral condition, but no such difference with regard to Stroop congruence was exhibited by the low-fitness group.

### Behavioral data

Our behavioral results showing worse performances in the incongruent condition relative to the neutral condition are consistent with those of previous studies (Qiu et al., 2006; Fan et al., 2014; Chang et al., 2015) and suggested a typical “Stroop effect” (Stroop, 1935). In contrast with the neutral condition, which involves predominantly chromatic information, the incongruent condition involves conceptual discrepancies between the semantic meanings of the words and the chromatic dimensions (MacLeod and MacDonald, 2000). According to the parallel distributed processing of the Stroop Test (Cohen et al., 1990), conflict arises because the tendency of reading out the semantic meaning is stronger than that of naming the color. To perform the incongruent condition successfully, increasing attentional inhibitory processing engagement is required to inhibit the task-irrelevant information (MacLeod, 1991; Nigg, 2000); that is, greater cognitive resources are required of the participant in order for him or her to disengage their attention away from the task-irrelevant information (Guiney and Machado, 2013).

Our results indicating that the adults with higher levels of cardiorespiratory fitness demonstrated shorter response times across both the neutral and incongruent Stroop conditions than those with lower cardiorespiratory fitness were also in line with those of several previous studies (Prakash et al., 2011; Weinstein et al., 2012; Gauthier et al., 2015). These findings suggest that a positive association between fitness and general cognitive functions is reflected by the Stroop Test, at least in late-middle-aged adults. The beneficial effect of higher fitness was further confirmed by no difference in accuracy levels, indicating that the shorter response times were not attributable to a speed-accuracy trade-off. This better cognitive performance associated with higher fitness levels might be associated with increased brain function in older adults. For example, aged adults with higher cardiorespiratory fitness levels have demonstrated not only better behavioral performances in the Stroop Test, but also increased recruited activation of the prefrontal and parietal cortices (Prakash et al., 2011), greater preserved volume of the right inferior frontal gyrus and precentral gyrus (Weinstein et al., 2012), as well as aortic elasticity (Gauthier et al., 2015). Our study also expands upon previous studies that typically employed the Erikson flanker task (Colcombe et al., 2004; Hillman et al., 2006). Taken together, our study again confirms that cardiorespiratory fitness is positively linked to cognitive functioning in the aged adults.

### ERD/ERS data

The novelty of the current study was its exploration of the association between cardiorespiratory fitness and cognitive performance through variations in alpha frequency band activity (Pfurtscheller and Lopes Da Silva, 1999). EEG activities, such as ERD and ERS, have been suggested to be reflections of the uncoupling and coupling of functional networks in the cortex (Bastiaansen and Hagoort, 2006). Additionally, upper and lower alpha frequencies have been linked primarily to attention and task-related processing, respectively (Klimesch et al., 2007). Examining the upper and lower alpha ERD/ERS of neuronal activity for a given function might thus provide alternative explanations for the influence of cardiorespiratory fitness on cognitive performance.

Our results revealed that the adults with high-fitness exhibit less upper alpha ERD, reflecting increased alpha synchronization, which has typically reflected reduced information processing in the “idling” state (Klimesch et al., 2007). According to the inhibition-timing hypothesis (Klimesch et al., 2007), however, enhanced cognitive performance is associated with increased upper alpha ERS through greater upper alpha activity or alpha oscillations, suggesting higher efficiency in inhibiting non-essential or task-irrelevant processing (Klimesch et al., 2007; Buschman et al., 2012; Bazanova and Vernon, 2014). Increased upper alpha frequency has also been observed during the encoding and retention interval periods, implying that inhibition of the retrieval of interference information was involved (Klimesch et al., 2005). That is, alpha synchronization could be considered to be a specific type of inhibition, in which the cognitive process is focused on the task-relevant information and interference from the non-task-relevant information is prevented (Klimesch et al., 2007). Accordingly, along with the positive association between fitness and Stroop Test performance, the results regarding higher upper alpha ERS might suggest that late-middle-aged adults with high levels of fitness have superior ability to inhibit or deselect the current task-irrelevant processing during the performance of the given task. Moreover, since no difference was observed in the upper alpha ERD values for the early epoch window between the high- and low-fitness groups, it is possible that a stronger influence of cardiorespiratory fitness occurs during the later stage of semantic encoding processes.

Lower alpha frequency desynchronization has been linked to attentional engagement for task-independent and non-stimulus factors (Klimesch et al., 1998; Klimesch, 1999). For instance, a decrease of lower alpha frequency activity, representing increased ERD, after a warning signal was observed, implies an association between lower alpha ERD and attentional demands such as alertness or expectance (Klimesch et al., 1998). In line with the interpretation of attentional processes, research involving dyslexic individuals observed significantly larger lower alpha desynchronization during the reading of words and pseudowords in such individuals than in non-dyslexic counterparts, suggesting that more attentional resources are engaged by dyslexic individuals during the encoding of words and pseudowords (Klimesch et al., 2001). Furthermore, during a combined Stroop-Task-Switching paradigm study, a larger degree of lower alpha ERD during the color-to-word condition compared to the word-to-word condition also suggested the involvement of attentional resources (Wu et al., 2015). That is, the larger positive lower alpha ERD values observed during the early epoch window following the stimulus onset observed in high-fitness individuals might suggest their superior ability, compared to those with lower cardiovascular fitness levels, to engage a greater amount of attentional resources in response to a stimulus.

In line with prior findings in which more alpha power for the incongruent condition compared to the neutral condition was observed (Hanslmayr et al., 2008), a larger lower alpha ERD in the incongruent condition than in the neutral condition in high-fitness individuals was observed in this study. However, it should be noted that during the later epoch window, no lower alpha ERD difference between the incongruent and neutral conditions was observed in the low-fitness participants. Based on the parallel distributed processing account of the Stroop effect (Cohen et al., 1990), conflict arises when participants attempt to inhibit the highly activated, yet task-irrelevant response tendency (i.e., reading the word), and selectively attend to the relevant, but less activated response tendency (i.e., naming the color) in the incongruent condition. That is, increasing attentional inhibitory processing engagement is required to inhibit the task-irrelevant information (MacLeod, 1991; Nigg, 2000). Considering the superior behavioral performance of the high-fitness individuals compared to their low-fitness counterparts, the current findings might imply that high-fitness late-middle-aged adults are able to utilize more attentional resources for conflict resolution than their low-fitness older counterparts.

### Limitations and future directions

Despite novel findings associated with cardiorespiratory fitness levels and cognitive functioning from the perspective of alpha ER% values, some limitations of the present study should be considered. First, a previous study reported differences in neuroelectric activities between males and females (Shen, 2005). Given that only male participants were recruited, whether or not our findings can be generalized to both genders requires further examination. Second, the association between cardiorespiratory fitness and executive control might be affected by other factors, such as education level and social economic status, in which these confounding factors are warranted to control. Additionally, while the Stroop Test, being a classical neuropsychological assessment, reflects inhibition appropriately, it should be noted that several different processes associated with inhibition can be involved during various inhibitory tasks, such as motor response inhibition tasks (e.g., the stop-signal task and go/no-go task) and the interference aspect of inhibition tasks (e.g., the Erickson flanker task and Stroop Test; Wostmann et al., 2013). Examining the alpha frequencies induced by utilizing various inhibitory tasks might provide a more comprehensive map for the relationship between fitness and inhibition. Moreover, the current alpha ERD/ERS study focused solely on the variation of alpha frequencies on the Fz electrode location. While it is reasonable as a preliminary study because of the high association between the prefrontal cortex and executive control, including more electrodes as well as more EEG frequency bands might provide more comprehensive information related to ERD/ERS and clarify the associations between various cortical regions during cognitive processing. Future study is also encouraged to utilize longitudinal design to establish causal relationship.

## Conclusion

The findings reported herein add imperative knowledge to the fitness-cognition literature by exploring differences in alpha ERD/ERS activity between high-fitness and low-fitness middle-aged adults. Middle-aged adults with greater cardiorespiratory fitness were showed superior cognitive function, as reflected by the Stroop Test, in comparison to those with lower fitness. Additionally, the results of neuro-electrophysiological index of the alpha ERD/ERS suggested that cardiorespiratory fitness is also related to the efficiency of inhibiting task-irrelevant information processing as well as the ability to engage greater attentional resources to given cognitive demands. Future research is encouraged to consider that generalization with regard to gender, types of inhibition cognitive tasks, and EEG frequencies to further the understanding of the relationships between cardiorespiratory fitness and cognitive functioning in different populations.

## Author contributions

YC, TH, and CC made substantial contribution to conceive and design the experiment protocol, organize the experiment, as well as critically revise manuscript; KY, TS, and JL involved in conducting the experiment, statistical analysis processes, and development of overall research plan; all authors have involved in part of writing and approved the final manuscript.

### Conflict of interest statement

The authors declare that the research was conducted in the absence of any commercial or financial relationships that could be construed as a potential conflict of interest.
